# Positioning analysis of Spanish politicians through their Twitter posts versus Spanish public opinion

**DOI:** 10.1057/s41599-023-01805-9

**Published:** 2023-06-08

**Authors:** Azucena Penelas-Leguía, Estela Nunez-Barriopedro, Jose María López-Sanz, Rafael Ravina-Ripoll

**Affiliations:** 1 Universidad de Alcalá, Alcalá de Henares, Spain; 2 Universidad de Cádiz, Cádiz, Spain

**Keywords:** Business and management, Information systems and information technology

## Abstract

The evolution of Information and Communication Technologies (ICT) has changed the way we communicate. Access to the Internet and social networks has even changed the way we organise ourselves socially. Despite advances in this field, research on the use of social networks in political discourse and citizens’ perceptions of public policy remains scarce. So, the empirical study of politicians’ discourse on social networks in relation to citizens’ perception of public and fiscal policies according to their political affinity is of particular interest. The aim of the research is, therefore, to analyse positioning, from a dual perspective. Firstly, the study analyses the positioning in the discourse of the communication campaigns posted on social networks of Spain’s most prominent politicians. And secondly, it evaluates whether this positioning is reflected in citizens’ opinions about the public and fiscal policies being implemented in Spain. To this end, a qualitative semantic analysis and a positioning map is performed on a total of 1553 tweets published between 1 June and 31 July 2021 by the leaders of the top ten Spanish political parties. In parallel, a cross-sectional quantitative analysis is carried out, also through positioning analysis, based on the database of the Public Opinion and Fiscal Policy Survey of July 2021 by the Sociological Research Centre (CIS), whose sample is 2849 Spanish citizens. The results show a significant difference in the discourse of political leaders’ social network posts—which is more pronounced between right-wing and left-wing parties—and only some differences in citizens’ perception of public policies according to their political affinity. This work contributes to identifying the differentiation and positioning of the main parties and helps to guide the discourse of their posts.

## Introduction

With the development of technology and digitalisation, there is growing interest in research on marketing strategy that considers the integration of online and offline contexts (Alonso-Garcia et al., 2023) and social change (Cuesta-Valiño et al., 2022). For this, it is started from a context where the growing mediatisation of politics makes social networks increasingly influential in political communication campaigns (Kelm et al., 2019). The effect of politicians’ posts has been studied on Facebook recently by some authors (Abejón et al., 2019; Acosta, 2012). However, of all social networks, Twitter is the one that, as a medium for political communication, has received the most attention in the most recent literature (Ernst et al., 2019; Hameleers, 2022; Kelm et al., 2019; Kulshrestha et al., 2018; Romero-Rodríguez et al., 2015).

Most studies concerning political communication are based on a qualitative research design (Abejón et al., 2019; Acosta, 2012; Acosta et al., 2017; Ernst et al., 2019; Kelm et al., 2019; Kulshrestha et al., 2018; Rojas-de-Gracia, 2019; Romero-Rodríguez et al., 2015), with quantitative studies being in the minority (Garmendia et al., 2022; Lorenzo-Rodríguez and Torcal, 2022; Nemčok, 2020). One of the novelties of this research is the study of political positioning from a double perspective, both qualitative and quantitative. On the one hand, we analyse the positioning in the discourse of the Twitter communication campaigns of the main politicians in Spain through their posts. On the other hand, a quantitative analysis is carried out based on data on citizen opinion regarding public and fiscal policies carried out in Spain, published by the Centro de Investigaciones Sociologicas (CIS), according to their political affinities. Both studies are carried out in the period between 1 June and 31 July 2021. The intention is to use a similar timing for the qualitative and quantitative studies in order to be able to make a parallel analysis.

Another feature of this study is that it considers a broad spectrum of political parties, both in the quantitative part, where the opinions of citizens of ten political parties are analysed, and in the qualitative part, where the messages disseminated on the personal Twitter accounts of eleven political leaders are analysed. In the case of the ERC party, not only the posts of its leader were analysed, but also those of its representative in Congress, due to the peculiar situation of the former, who was in prison during the period analysed.

Recent studies claim that social media act as an instrument to increase self-esteem by contributing to dissemination of political ideas (Bail, 2021; Cuesta-Valiño et al., 2023b). As a result, there has been a growing interest in the literature on party positioning (Doyle, 2011; Ivarsflaten, 2008; Lubbers et al., 2002; March and Rommerskirchen, 2014; Rooduijn, 2018; Schulze et al., 2020; Van Hauwaert and van Kessel, 2018; Visser et al., 2014). However, research from the societal perspective on public and fiscal policies has been scarce.

This paper aims to address this gap in the literature and contribute to knowledge about the positioning from a double prism. On the one hand, from the discourse of political leaders’ of the main political parties and on the other from the side of the public opinion of citizens as voters.

To this end, based on the analysis of politicians’ statements on social networks, this work contributes to understanding the positioning of the discourse, through a factor analysis of correspondences of the terminology most used by each of the leaders for a greater political differentiation and ideological positioning in social network. As previous studies have argued (Schulze et al., 2020) if differentiation becomes too extreme, it is likely to lead to political conflict, thus hindering consensus in the proper functioning of the democratic political system (DiMaggio et al., 1996; Hetherington and Rudolph, 2015; Iyengar et al., 2019).

On the other hand, in times of rising inflation and loss of purchasing power, the study of public service management and fiscal policy is of great importance to academics, practitioners and the general public (Núnez-Barriopedro et al., 2023). The growing needs for public revenue in developed countries, including Spain, have led to an expansion of public spending to cover the commitments made by the state (Goenaga Ruiz de Zuazu, 2018). So, from the perspective of citizenship, this work contributes to knowledge about the positioning from the perspective of welfare perceived through the payment of taxes (Novo et al., 2020). Specifically, in aspects that directly affect them, such as the use of their taxes and satisfaction with public services.

In accordance with the statement of purpose of the research, this study proposes the following research questions (RQ):

RQ1. How do politicians position themselves through their social media posts?

RQ2. Are there significant differences in citizens’ opinions on the degree of satisfaction with the performance of public services, depending on the political party to which they are aligned?

RQ3. Are there significant differences in citizens’ opinions on tax policy, depending on the political party to which they are aligned?

This double prism allows to analyse whether there is a parallelism in the positioning of politicians in social networks from the language of political leaders and the political positioning outside the networks from the public opinion of citizens.

## Theoretical framework

The evolution of Information and Communication Technologies (ICT) has changed the way we communicate, express ourselves and interact in different areas. In particular, access to the Internet and social networks has even changed the formulas of social organisation (Castillo-Abdul et al., 2022; Romero-Rodríguez et al., 2015). It is more complex to maintain control of information, creating a space for the extension of independent public debate (Etling et al., 2014).

In recent years, all political parties have used social networks as an important communication mechanism (Barberá, 2020; Jungherr, 2016; Lorenzo-Rodríguez and Torcal, 2022). Thus, in contemporary democracies, political leaders tend to behave strategically in social networks (Garmendia et al., 2022; Gibson, 2015). Specifically, in Spain there has been a strong increase in partisan Twitter, being one of the 10 countries in the world (35%) that most use this type of social networks (Newman et al., 2020). As a result, they often express their ideology in the content of their tweets, while emphasising their beliefs on Twitter (Engesser et al., 2017; Ernst et al., 2019; Kriesi, 2018).

Although social networks appear as decentralised channels of communicative flow, they continue to maintain epicentres of attention on opinion leaders, as they have greater penetration and distribution nodes for messages, which is why they are established as emitters of ideology that is consumed, replicated and shared (Romero-Rodríguez et al., 2015). Thus, networks function as groups organised around socially shared values and principles that reveal a polarised structure of some versus others (Acosta, 2012).

Several studies maintain that social media tend to produce homogenous, self-reinforcing exposure to the most extreme radicals, thus over-representing levels of disagreement and activating social identities (Bail, 2021). Furthermore, social media act as an instrument for increasing self-esteem (Cuesta-Valiño et al., 2023a).

Therefore, the following hypothesis is put forward for the perspective of politicians in Spain:

Hypothesis 1 (H1a). Positioning among Spanish political leaders with respect to the most frequently used terms in their communications posted on Twitter is significantly different.

Extreme political positions fuel distrust of traditional parties and media. Both parties and voters have become increasingly polarised, and divisions between political camps appear to be deepening (Galston, 2018; Iyengar et al., 2019). The phenomenon of “political polarisation” in Spain has recently raised interest in public debate (Torcal and Carty, 2022).

From a sociological perspective, polarisation is “a social phenomenon that occurs when individuals align their beliefs in extreme and conflicting positions” (Isenberg, 1986; Sunstein, 2002, p. 175). Likewise, polarisation is a discursive strategy in which the mechanism of positive self-presentation of some is developed, as opposed to the negative presentation of others (Schulze et al., 2020; Van Dijk, 2003). From a cognitive point of view, the polarised person reduces his or her perception of the rival group to stereotypes, to simplistic and rigid categories, which contain a minimal group identification and a strong negative moral characterisation (Abejón et al., 2019).

In previous studies there are discrepancies regarding its intensity and causes of polarisation, the focus of attention being the ‘polarising/polariser tone’ of many of the slogans of political representatives and the impact they generate in social networks (Torcal and Magalhães, 2022).

These trends appear to be fuelled, in part, by digital communication: populist actors use online media very efficiently to spread their messages (Engesser et al., 2017; Gerbaudo, 2017; Stier et al., 2017), and (hyper)partisan media reinforce radical and anti-democratic ideas through repetition on various social networks (Prior, 2013; Starbird, 2017), although other recent studies do not confirm this (Lorenzo-Rodríguez and Torcal, 2022).

Polarisation can be seen as threats to the liberal democratic order (Lozada, 2004; Schulze et al., 2020). Stereotypical perceptions of others hinder dialogue and the possibilities of reaching agreements based on the debate of ideas.

There are several studies that focus on the polarisation of Spanish society (Garmendia et al., 2022; Miller, 2021; Torcal and Comellas, 2022). Others focus on what is called “affective polarisation” (Orriols and León, 2021; Rodríguez et al., 2022; Rodríguez-Teruel, 2021; Simón, 2020), which argue that Spain shows high and increasing levels of affective polarisation (Gidron et al., 2020; Torcal and Comellas, 2022). Además, Spain, presents two different polarising conflicts: the partisan one and the emerging centre-periphery one based on the activation of different territorial/national identities (Lorenzo-Rodríguez and Torcal, 2022).

In previous studies there are discrepancies regarding the intensity and causes of polarisation, the focus of attention being the ‘polarising/polariser tone’ of many of the slogans of political representatives and the impact they generate in social networks (Torcal and Magalhães, 2022). Therefore, the following hypothesis is proposed:

Hypothesis 1 (H1b). Positioning among Spanish political leaders with respect to the most frequently used terms in their communications posted on Twitter is polarised between right-wing and left-wing parties and polarised between constitutionalist and pro-independence.

Citizens search the Internet for information on the social and political issues that interest them on those websites or social networks that are most in line with their ideology, in order to reaffirm their beliefs and avoid inconsistencies (Díaz, 2015; Iyengar et al., 2019). This different position, that can be seen between the different political parties, in their communications and in what they express on their social networks, is transferred to the general population that follows the different political parties and reads their comments on social networks (Schuliaquer and Vommaro, 2020). So, citizens have opted to use a hybrid communication system, where analogue and digital media, such as social networks, are interrelated (Chadwick, 2013)

However, it is necessary to check the response of citizens in terms of their satisfaction with public services. According to the Instituto de Estudios Fiscales (2020), healthcare receives the highest level of satisfaction. Other studies point out that ideology, especially the left/right dichotomy, is very important when analysing attitudes towards welfare policies, especially health and education (Blekesaune and Quadagno, 2003), varying the perception of satisfaction with these policies (González and Carreras, 2012). Welfare state provisions and public services are seen by citizens as an acquired right. This may lead to a greater rejection of reforms, with a consequent reduction in the services provided.

So, the following hypothesis is put forward for the perspective of voters in Spain:

Hypothesis 2 (H2a). Citizens’ opinions on the functioning of public services shows a significantly different positioning depending on the political party to which they are aligned.

Traditionally, Spanish politics has been articulated along the left-right dimension (Torcal et al., 2007), which includes economic and cultural issues that are strongly aligned in the case of Spain (Rovny and Polk, 2019). However, in recent years, it has been asserted that the climate of polarisation has also increased markedly among the citizens (Torcal and Comellas, 2022).

Additionally, another topic of interest in the literature is that of the different dimensions that can shape partisan polarisation. Thus, they point out that partisan polarisation is linked to a redefinition of conflicts with respect to other identities present in the political and social sphere, such as the regionalist/nationalist identity conflict (Garmendia et al., 2022; Torcal and Carty, 2022). Therefore, the following hypothesis is proposed:

Hypothesis 2 (H2b). Citizens’ opinions on the functioning of public services are polarised between voters of right-wing and left-wing parties and polarised between constitutionalist and pro-independence.

With regard to the allocation of economic resources, few studies have focused on the opinion on their use in Spain according to political ideology. According to the BBVA Foundation survey (2013), most Spaniards are inclined to increase public spending on healthcare, care for the disabled and elderly, help for the unemployed, education and scientific research. Although, there are also differences depending on the ideology of each citizen. According to Del Pino (2007), Spaniards do not share the idea that to improve the quality and quantity of Welfare State services, it is necessary to increase the tax burden. But rather that existing resources should be managed better and more clearly. This idea is more marked among citizens who call themselves “right-wing”. For Miller (2020), there is a clear differentiation around fiscal policies, especially on issues related to tax redistribution. Therefore, the following hypothesis is proposed:

Hypothesis 3 (H3a). Citizens’ opinions on the allocation of resources to public services show a significantly different positioning depending on the political party to which they are aligned.

Regarding the use of public resources, some authors find an ideological polarisation between left and right-wing voters, but it is greater on issues such as immigration than on public health or public services (Miller, 2020). This polarisation has been growing over the years. Until recently, this term was only used by political scientists because the positions of right-wing and left-wing voters on this issue were very homogeneous (Barreda, 2021). After the COVID-19 pandemic, this polarisation on the use of public resources was increased in certain respects, especially on the measures that should be taken (Miller, 2021). Therefore, the following study hypothesis is proposed.

Hypothesis 3 (H3b). Citizens’ views on the government’s commitment of resources to public services are polarised between voters of right-wing and left-wing parties and polarised between constitutionalist and pro-independence.

## Methods

The research design was an exploratory study with an in-depth review of the literature, followed by a correlational study with a double perspective of qualitative and quantitative analysis. The aim is to analyse the social network positioning of Spain’s main political leaders, and whether this is reflected in the positioning of their parties’ sympathisers. To this end, the study analyses the top ten Spanish political parties, according to the number of seats won in the last general elections in Spain on 10 November, 2019.

In the qualitative analysis, a semantic analysis has been carried out followed by an analysis of the positioning of the political leaders (Multiple Correspondence Factor Analysis) with respect to the tweets published on their Twitter accounts, between 1 June and 31 July 2021. A total of 1553 tweets posted by the eleven political leaders in Spain are analysed. Twitter’s API has been used to scrape these tweets. The statistical analysis technique based on the semantic information analysed from each of the tweets published by each of the political leaders was the creation of positioning maps, using Le Sphinx software.

The correspondence between the political parties, the political leaders they represent and the distribution of tweets published on their own Twitter accounts is shown in Table 1. Eleven political leaders representing a total of ten represented parties are analysed. The number of leaders selected is higher, due to the fact that some of these parties have had significant incidents with their leaders in the period analysed. Specifically, in “ERC-Soberanistas” their leader “Oriol Junqueras” was in prison until 23 June, 2021, so “Gabriel Rufián”, spokesman of the ERC group in Congress, has been added to the study. Likewise, on the date of the study, in the political party “Podemos”, Ione Belarra had already been elected as the new secretary general of Podemos, replacing Pablo Iglesias.

**Table 1:** Sample information used in the qualitative analysis.

Political party	Political leaders	Original tweets	Retweets	Total tweets
PSOE	Pedro Sánchez	233	181	414
PP	Pablo Casado	247	43	290
VOX	Santiago Abascal	81	243	324
PODEMOS-UP	Ione Belarra	244	202	446
ERC-SOBERANISTAS	Oriol Yunqueras	31	93	124
	Gabriel Rufián	102	391	493
CS	Inés Arrimadas	193	170	363
JXCAT-JUNTS	Carles Puigdemon	107	94	201
PNV	Andonio Ortuzar	9	90	99
EH BILDU	Arnaldo Otegi	61	50	111
MAS PAIS	Iñigo Errejón	245	354	599
	TOTAL	1553	1911	3464

Source: Own elaboration based on the website of each party and the Twitter accounts of each political leader.

In the quantitative analysis, an analysis of voter positioning according to political affinity has been carried out by means of a correspondence factor analysis (CFA). With this analysis, it is possible to discover affinities between two sets of variables, presented in the form of a table, both of frequencies and mean values (Miquel et al., 1997). Furthermore, it provides many tools that can handle complex datasets and observations on different measurement scales can be coded to be analysed together (Greenacre, 2019). Correspondence factor analysis can be used in the study of the image and positioning of products and brands, private entities, public institutions, political leaders, etc. (Santesmases, 2009). The opinion of Spanish citizens is obtained through the database of the survey “Public Opinion and Fiscal Policy Survey” of July 2021 published by the Centro de Investigaciones Sociológicas (CIS), regarding the top ten Spanish political parties (2019 elections). The software used was SPSS version 27 and DYANE version 3.

The sample size is 2849 Spanish citizens. For a confidence level of 95.5% (two sigmas) and P = Q, the sampling error is ±1.9% for the whole sample. The procedure was simple random sampling by random selection of landline and mobile phones with a percentage of 28.3% and 71.7%, respectively. The selection of individuals was carried out by applying quotas for sex, age and proportional affiliation.

The fieldwork was carried out by the CIS between 21 and 29 July 2021, among the Spanish population of both sexes aged 18 and over. The questionnaires were administered by computer-assisted telephone interview (CATI).

Correspondence factor analysis (CFA) has been performed using the answers obtained by the CIS survey (2021).

1.- Positioning by political affinity, according to the degree of satisfaction with the functioning of public services. For this purpose, a CFA is carried out between the variable: voters’ political affinity with Q32. “Supposing general elections were held again tomorrow, i.e., to the Spanish Parliament, which party would you vote for?” as a categorical variable and each of the nine public services as scale variables (Education; Healthcare; Pension Management; Administration of Justice; Public Safety; Social Services; Public Transport; Public Works (roads, water treatment plants, etc.); Aid to Dependent Persons) corresponding to Q06. “To what extent would you say that each of the following public services function very, fairly, not very or not at all satisfactorily (scale of 1 very satisfactory, 5 not at all satisfactory)?”

2- Positioning by voters’ political affinity, according to the degree of satisfaction with the dedication of resources to public services. For this purpose, a CFA is carried out between the variable: voters’ political affinity, Q32. “Supposing general elections were held again tomorrow, i.e., to the Spanish Parliament, which party would you vote for?” as a categorical variable and each of the 15 public services and benefits as scale variables (Education; Public Works; Unemployment Protection; Defence; Public Safety; Health; Culture; Housing; Justice; Social Security/Pensions; Transport and Communications; Environmental Protection; Development Cooperation; Research in Science and Technology; Aid to Dependent Persons corresponding to Q08 “Do you think that too many (1), what is necessary (2) or too few (3) resources are devoted to each of the following public services”?.

## Results

The following sub-headings show the results of the correspondence factor analyses and the positioning maps corresponding to the qualitative and quantitative parts of the study, respectively. Sub-section “Analysis of the positioning of the publications of the main political leaders on Twitter” shows the qualitative analysis in which a semantic analysis was carried out followed by an analysis of the positioning of political leaders with respect to the tweets published on their Twitter accounts. Section “Analysis of voter positioning” shows the quantitative analysis in which the voters’ responses to the CIS survey have been analysed.

### Analysis of the positioning of the publications of the main political leaders on Twitter

In the Multiple Correspondence Factor Analysis, in which the degree of relationship and the positioning of the political leaders with respect to the words most mentioned in the posts published on their Twitter accounts is analysed, the relationship is highly significant, with a *P*-value = <0.01; Chi-square = 4208.0 and degrees of freedom = 1000. Table 2 shows the explanation of inertia, where axis 1 explains 29%, axis 2 17%, axis 3 around 10% each. Thus, the first five axes cumulatively account for 78% of the variance explained.

**Table 2:** Variance explained by the factors.

	F1	F2	F3	F4	F5
Eigenvalues	0.061	0.035	0.026	0.023	0.02
Contribution to inertia	28.57%	16.71%	12.29%	10.68%	9.63%

On the left side of axis 1, Pedro Sánchez, Ione Belarra and Iñigo Errejón are positioned, these leaders being the representatives of left-wing ideological parties, with Pedro Sánchez standing out as the current president of the government for the “PSOE” party who governs in coalition with the “PODEMOS-UP” party. However, on the right side of axis 1, the leaders Pablo Casado, Inés Arrimadas and Santiago Abascal are positioned, being the representatives of right-wing ideological parties, currently representing the opposition. Among the words that can be observed on the left side of axis 1 are country, woman, social, equality, recovery, and new society, which are common words in left-wing discourses in the political parties analysed. While on the right-hand side, government, court, law, plotter, Spaniard, and national stand out, being the usual words used in right-wing opposition speeches in the political parties analysed. Therefore, it can be seen that there is a clear left-right polarisation on axis 1.

In axis 2, there is the presence of pro-independence parties such as Junqueras, Gabriel Rufian, Andonio Ortuzar, Arnaldo Otegui, and Carles Puigdemont (KRLS), with the words independence, right, freedom, and able being the most prominent. However, in the lower part of axis 2, is the presence of the politicians Sanchez Castejon on the lower-left side with the words today, agreement, and love, also located on the left, and on the lower-right side Pablo Casado and Inés Arrimadas with the words unity, Spanish, and national on the right and with the words Spain, family centred at the bottom. Therefore, it can be seen on axis 2 that there is a clear pro-independence-nationalist polarisation.

**Fig. 1:**
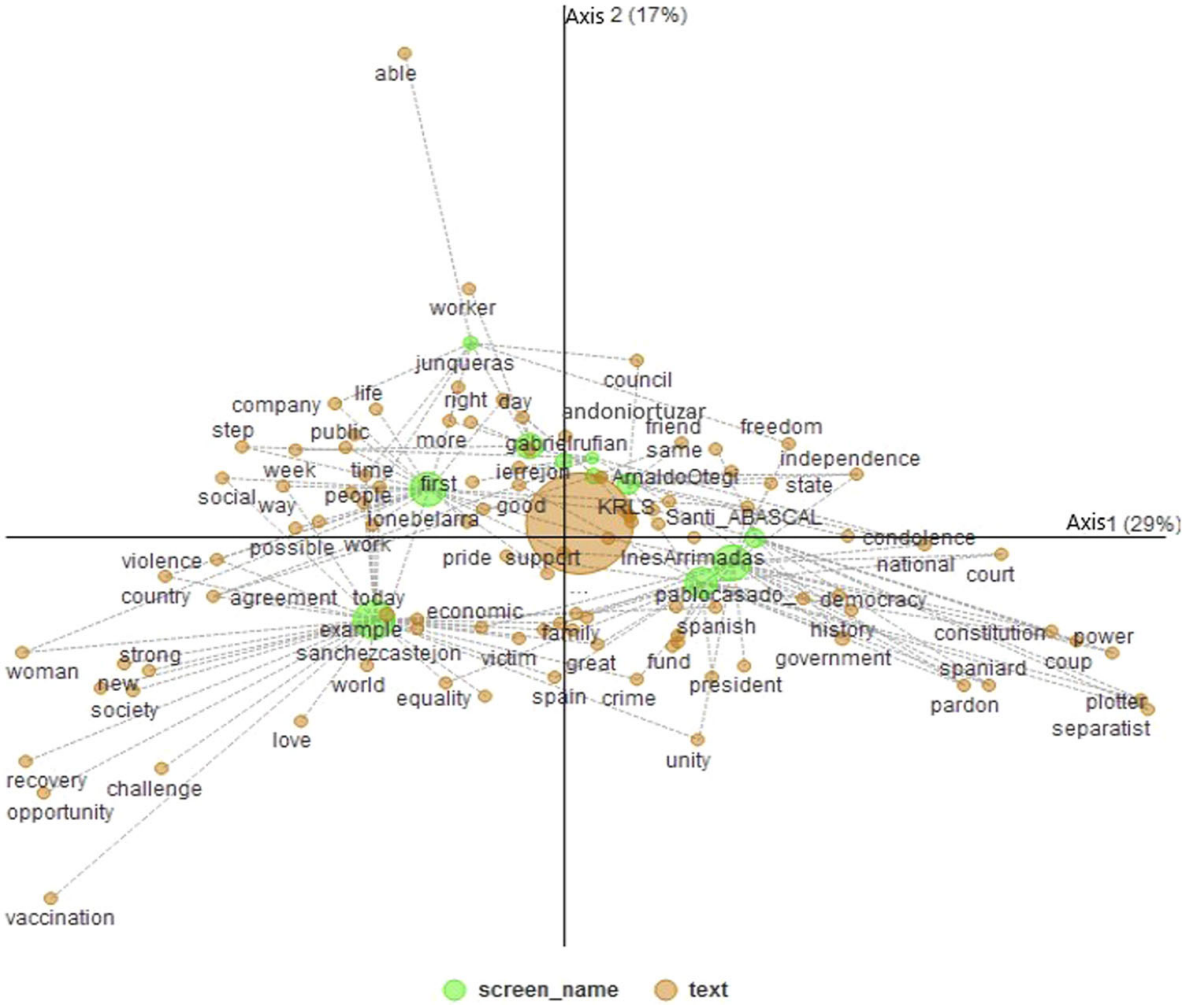
Map of the positioning of political leaders with respect to the most mentioned words in the posts published on their Twitter accounts. Source: Own elaboration.

As shown in Fig. 1 and Tables 2 and 3 (see supplementary material), respectively, hypotheses H1a and H1b can be accepted, because political leaders are clearly positioned in a polarised way in correspondence with their political ideologies, their discourse being consistent with their ideology.

**Table 3:** Column and row coordinate elements (see supplementary material).

Column	AXIS 1			AXIS 2		
screen_name	Coordinate	Correlation	% Inertia explained	Coordinate	Correlation	% Inertia explained
andoniortuzar	0.05	0.001	0.02%	0.226	0.024	0.77%
ArnaldoOtegi	0.052	0.006	0.13%	0.174	0.066	2.39%
gabrielrufian	−0.002	0	0.00%	0.216	0.202	3.74%
ierrejon	−0.058	0.021	0.57%	0.261	0.419	19.51%
InesArrimadas	0.234	0.321	13.20%	−0.134	0.106	7.43%
ionebelarra	−0.233	0.395	14.90%	0.135	0.133	8.56%
junqueras	−0.161	0.021	0.82%	0.559	0.257	16.88%
KRLS	0.108	0.056	1.37%	0.15	0.108	4.50%
pablocasado_	0.289	0.527	25.15%	−0.077	0.037	3.05%
sanchezcastejon	−0.324	0.602	34.88%	−0.242	0.335	33.17%
Santi_ABASCAL	0.328	0.365	8.99%	−0.005	0	0.00%
Row						
text						
today	−0.304	0.424	2.41%	−0.226	0.235	2.28%
government	0.477	0.572	4.96%	−0.296	0.22	3.26%
country	−0.685	0.869	8.42%	−0.115	0.025	0.41%
spain	−0.014	0.001	0.00%	−0.408	0.791	4.74%
people	−0.342	0.546	1.65%	0.175	0.143	0.74%
law	0.316	0.24	1.20%	0.085	0.017	0.15%
year	0.065	0.047	0.05%	0.171	0.328	0.64%
day	−0.071	0.011	0.06%	0.344	0.269	2.28%
good	−0.079	0.027	0.08%	0.148	0.096	0.46%
right	−0.106	0.024	0.12%	0.395	0.339	2.97%
family	0.021	0.002	0.01%	−0.224	0.213	0.94%
great	0.059	0.01	0.04%	−0.313	0.288	1.80%
spanish	0.258	0.171	0.66%	−0.206	0.109	0.72%
work	−0.328	0.485	1.03%	0.032	0.005	0.02%
time	−0.317	0.453	0.91%	0.143	0.092	0.32%
thank	−0.345	0.432	1.03%	0.096	0.034	0.14%
freedom	0.385	0.337	1.19%	0.267	0.162	0.98%
support	−0.027	0.004	0.01%	−0.108	0.06	0.17%
more	−0.198	0.087	0.29%	0.333	0.245	1.38%
congratulation	−0.006	0	0.00%	−0.251	0.221	0.82%

### Analysis of voter positioning

For the positioning of the voters of the different political parties, the following correspondence factor analysis (CFA) has been undertaken with the answers obtained by the CIS survey (2021).

1.- Positioning by political affinity, according to the degree of satisfaction with the functioning of public services.

2.- Positioning by voters’ political affinity, according to the degree of satisfaction with the dedication of resources to public services.

**Fig. 2:**
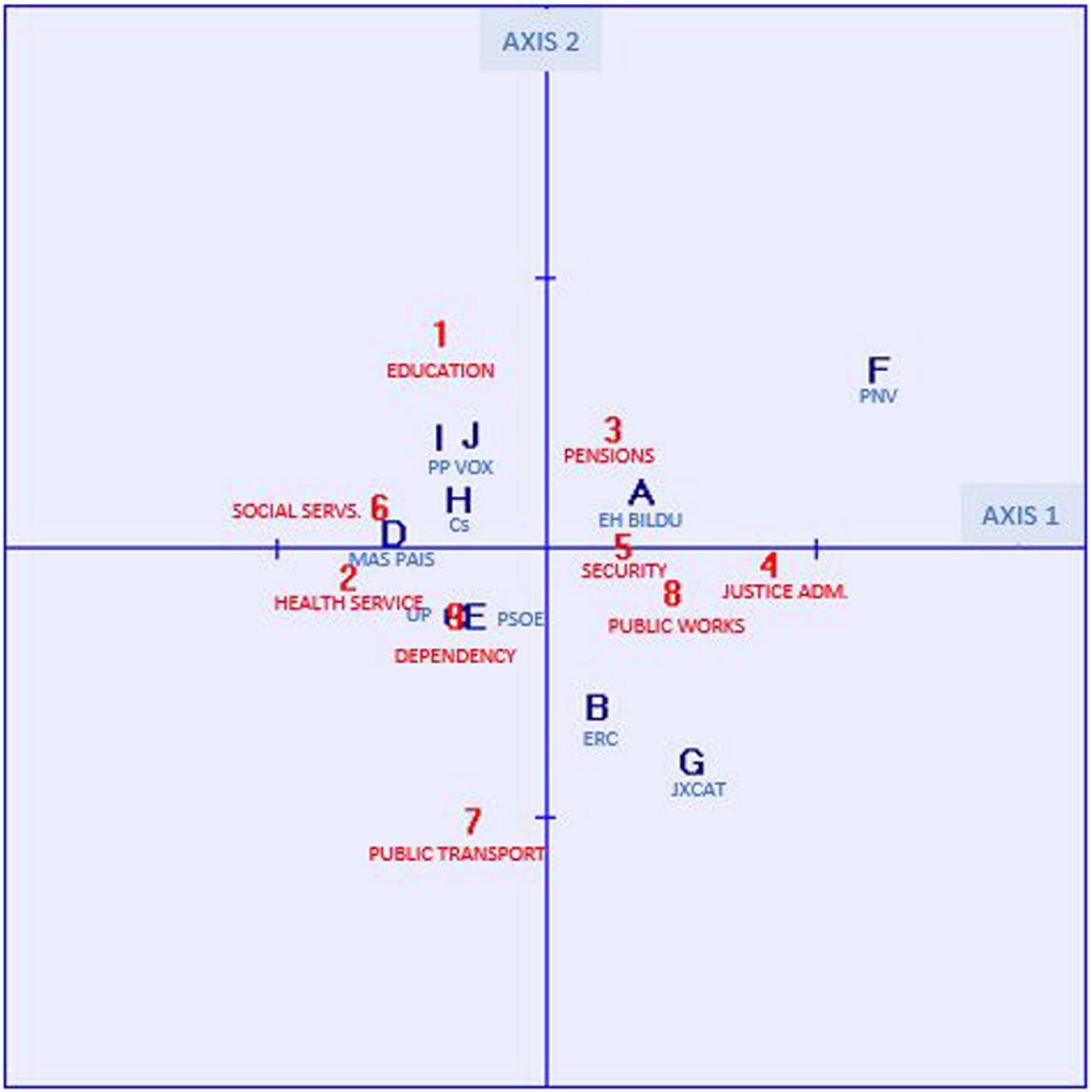
Map of voter positioning by political affinity, according to the degree of satisfaction with the functioning of public services. Source: Own elaboration based on Public Opinion and Fiscal Policy Survey, 2021.

CFA 1 is based on data on satisfaction with the functioning of public services, where 1 is very satisfied, and 5 not at all satisfied. Higher values should, therefore, be interpreted as showing greater dissatisfaction. The graph in Fig. 2 shows the positioning of voters and public services. The results obtained (Tables 4 and 5) show a high explanation of the inertia of the first two axes: axis 1 explains 46.39% of the inertia and axis 2, 34.29%, reaching a total of 80.68% of explained inertia for these two axes.

**Table 4:** Variance explained by the factors.

	Factor 1	Factor 2
Eigenvalues	0.0026	0.0019
Contribution to inertia	46.3893	34.2894

Total Inertia: 0.005670 Chi^2^: 1.5230.

**Table 5:** Column and row coordinate elements.

Column	AXIS 1			AXIS 2		
	Coordinate	Correlation	% Inertia explained	Coordinate	Correlation	% Inertia explained
EH Bildu	0.035	0.232	4.47%	0.022	0.090	2.36%
ERC	0.018	0.081	1.30%	−0.037	0.788	17.12%
Unidas Podemos	−0.034	0.464	4.68%	−0.024	0.225	3.07%
Más País	−0.057	0.671	12.59%	0.006	0.008	0.21%
PSOE	−0.027	0.490	2.55%	−0.024	0.402	2.83%
PNV	0.123	0.768	50.31%	0.066	0.224	19.82%
JxCat	0.053	0.302	11.68%	−0.078	0.650	34.05%
Ciudadanos	−0.034	0.361	4.23%	0.019	0.118	1.87%
PP	−0.037	0.328	5.21%	0.042	0.412	8.85%
VOX	−0.027	0.208	2.98%	0.043	0.508	9.82%
Row						
Education	−0.038	0.153	5.77%	0.080	0.661	33.77%
Health	−0.072	0.889	17.89%	−0.010	0.016	0.44%
Pensions	0.026	0.206	3.06%	0.045	0.629	12.64%
Justice Admin.	0.082	0.882	38.29%	−0.005	0.003	0.17%
Security	0.029	0.730	3.29%	0.002	0.002	0.01%
Social Services	−0.061	0.581	14.62%	0.016	0.038	1.28%
Public Transport	−0.027	0.056	2.43%	−0.100	0.787	46.39%
Public Works	0.047	0.707	9.18%	−0.016	0.081	1.43%
Dependency	−0.033	0.414	5.47%	−0.024	0.217	3.87%

Axis 1 is mainly related, on the positive side, to voter dissatisfaction with the administration of justice (4) and, on the opposite side, with health (2) and social services (6). With respect to voters, the relationship on the positive side of the axis with PNV voters (F) followed by JxCAT (G) stands out, while on the opposite side Más País (D) stands out.

Axis 2 is defined mainly on the positive side by the education variable (1) and on the opposite side of the axis by public transport (7). By political parties, the PNV (F) stands out on the positive side, followed by Vox (J) and PP (I). On the opposite side, JxCAT (G) and ERC (B).

That is to say, the biggest differences are provided by PNV voters due to their high degree of dissatisfaction with the administration of justice and satisfaction, especially with health and social services, as well as public transport. On the other hand, JXCat and ERC show greater dissatisfaction with public transport but satisfaction with education. In the rest of the parties, there are strong affinities between Vox and PP voters who are somewhat more dissatisfied with education, as well as between PSOE and UP, with a fairly centred position on the set of variables. However, there is no significant polarisation between left-wing and right-wing voters. The difference is greater between constitutionalist parties (on the left of axis 1) and pro-independence parties (on the right of axis 1), with the differences between the latter in terms of axis 2 between voters of Basque parties (PNV and EH Bildu) and Catalan parties (ERC and JXCat) being greater than those between constitutionalist voters.

Therefore, as shown in Fig. 2 and Tables 4 and 5, hypothesis H2a can be accepted as it shows a different positioning in the degrees of satisfaction with public services among voters of different political parties. However, the same cannot be said for hypothesis H2b, as there is no polarisation in this case between right-wing and left-wing voters, the differences being clearer between constitutionalist and pro-independence.

**Fig. 3:**
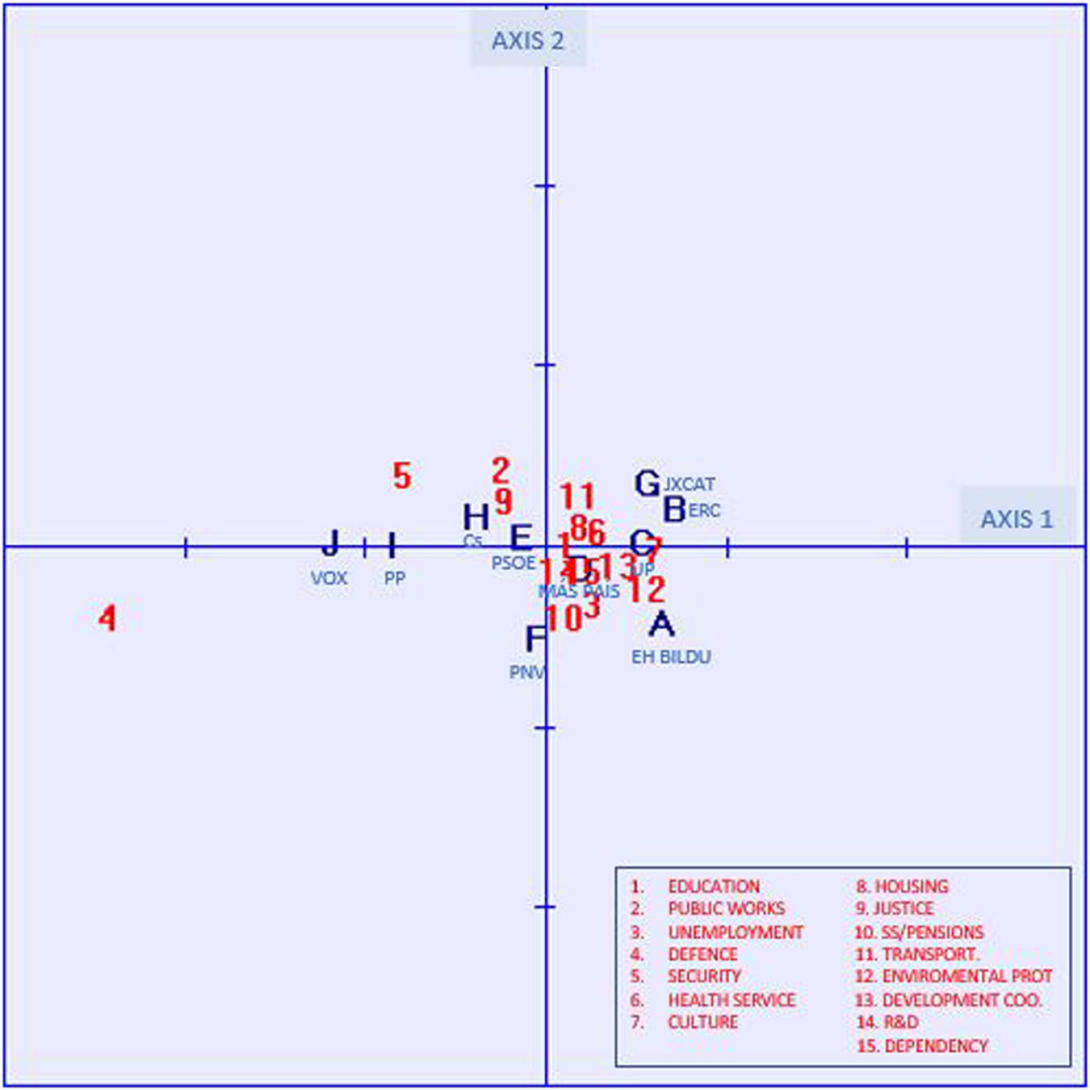
Map of voters’ political affinity, according to the degree of satisfaction with the dedication of resources to public services. Source: Own elaboration based on Public Opinion and Fiscal Policy Survey, 2021.

The graph in Fig. 3 shows the positioning derived from the second CFA regarding the dedication of resources to public services. The results obtained (Tables 6 and 7) indicate a significant explanation of 77.38% of the inertia of axis 1 related mainly to defence spending (4) (70.06% of the explained inertia of the axis), followed by security spending (5). The inertia explained by the rest of the variables is not significant. The voters closest to the opinion that too little is spent on defence (4) and security (5) are VOX (J) and PP (I), with ERC (B) and EH Bildu (A) in the opposite position. Axis 2, with an explained inertia of 12.92%, shows no significant differences between voters.

**Table 6:** Variance explained by the factors.

	Factor 1	Factor 2
Eigenvalues	0.0038	0.0006
Contribution to inertia	77.3787	12.9216

Total Inertia: 0.004875 Chi^2^: 1.8353.

**Table 7:** Column and row coordinate elements.

Column	AXIS 1			AXIS 2		
	Coordinate	Correlation	% Inertia explained	Coordinate	Correlation	% Inertia explained
EH Bildu	0.064	0.639	10.55%	−0.040	0.248	24.54%
ERC	0.070	0.833	13.10%	0.023	0.090	8.44%
Unidas Podemos	0.052	0.929	7.43%	0.003	0.004	0.17%
Más País	0.017	0.281	0.80%	−0.010	0.104	1.78%
PSOE	−0.013	0.441	0.48%	0.007	0.118	0.77%
PNV	−0.006	0.010	0.08%	−0.049	0.699	36.06%
JxCat	0.055	0.597	8.12%	0.037	0.277	22.58%
Ciudadanos	−0.041	0.673	4.30%	0.08	0.139	5.33%
PP	−0.082	0.983	17.81%	0.003	0.001	0.10%
VOX	−0.119	0.974	37.32%	0.004	0.001	0.22%
Row						
Education	0.011	0.399	0.24%	0.003	0.029	0.10%
Public Works	−0.024	0.194	0.85%	0.045	0.674	17.74%
Unemployment	0.025	0.282	1.14%	−0.030	0.400	9.69%
Defence	−0.242	0.973	70.06%	−0.038	0.024	10.45%
Security	−0.079	0.715	9.82%	0.042	0.196	16.10%
Health	0.028	0.580	1.54%	0.010	0.077	1.21%
Culture	0.060	0.840	6.69%	0.000	0.000	0.00%
Housing	0.019	0.185	0.66%	0.013	0.085	1.82%
Justice	−0.023	0.323	0.99%	0.027	0.437	8.03%
SS/Pensions	0.005	0.016	0.05%	−0.038	0.829	15.91%
Transport	0.013	0.111	0.28%	0.030	0.579	8.75%
Environmental Protection	0.051	0.717	4.94%	−0.022	0.136	5.62%
Development Coop.	0.035	0.868	2.34%	−0.010	0.063	1.02%
R&D	0.001	0.002	0.00%	−0.012	0.261	1.75%
Dependency	0.014	0.497	0.40%	−0.012	0.374	1.80%

As shown in Fig. 3 and Tables 6 and 7, the positioning of voters by political affinity on the dedication of resources to public services allows us to validate hypothesis H3a, with the defence expenditure variable clearly showing a different positioning of the VOX and PP parties. However, hypothesis H3b cannot be validated because, although they show a position ranging from right-wing parties (negative side of the axis 1) to left-wing parties (positive side of the axis 1), we cannot speak of polarised positions between left and right. Again, highlighting greater differences between the opinions of constitutionalist and pro-independence.

## Conclusions

The evolution of Information and Communication Technologies (ICT) has changed the way we communicate, express ourselves and interact in different areas. In particular, access to the Internet and social networks. In recent years, all political parties have used social networks as an important communication mechanism, and in Spain there has been a strong increase in partisan Twitter.

The data of the study performed on the discourse of politicians published on their social networks show a clear differentiation in their positioning between right-wing and left-wing parties, and pro-independence and nationalist parties. Political leaders use their social networks to communicate a polarised ideology that reinforces their political agenda and ideological positioning. Given the potential of social media to disseminate the opinion of political leaders with confirmation bias in the context of social support from like-minded followers, political dialogue can be hindered (Hameleers, 2022). Therefore, voter analysis outside the online environment can give a more realistic picture of the social context.

The positions of the voters of the different political parties analysed, in aspects such as the functioning of public services or the distribution of public resources among them, show only a few differences. It is significant in defence spending, showing different opinions in right-wing parties such as VOX and PP versus pro-independence parties. Opinions are slightly different between voters of constitutionalist and pro-independence parties in the valuation of public services. However, there are also some differences between the pro-independence parties themselves. These results are in line with research by Torcal and Comellas (2022) and could suggest a greater polarisation over territorial identities, especially with Catalonia.

The different positioning of politicians in social networks between right-wing and left-wing parties is clear, but it is not clearly reflected outside the social media. When analysing the positioning of political parties on the basis of their leaders’ speeches on social networks and comparing them with the opinions of their voters regarding public services, they do not show such polarised positioning between the left and right-wing blocs as one might think.

The data offered by voter opinion in a situation outside the social networks, such as the CIS survey, indicate some differences, but much less polarised than in the politicians’ messages on the Twitter social network being in line with Lorenzo-Rodríguez and Torcal’s (2022) experiment, where the results show that exposure to candidates’ Twitter accounts by self-selection does not increase affective polarisation. There are only some variations between left-wing and right-wing parties, most notably between constitutionalist and pro-independence. With regard to the two main political parties, PSOE and PP voters do not show such polarised positions as in the politicians’ messages on Twitter.

One of the limitations of this study is that it is a cross-sectional study in which online information published on Twitter by political leaders and offline information through public opinion in the CIS surveys of citizens is studied in parallel and in the same period of time, so that future work could consider a longitudinal study with panel data. Another limitation is the consideration of Spain as the geographical scope of this study, so that future studies could include more countries for a larger geographical scope. Finally, future studies could also add more variables to measure the positioning in other items.

## Supplementary information

Supplementary material_Table 3

Questionnaire

Dataset 1

Dataset 2

## Data Availability

Datasets were derived from public resources. The data of the study are extracted from the Twitter accounts post by political leaders in the qualitative analysis. And the database of the survey “Public Opinion and Fiscal Policy Survey” of July 2021 is published by the Centro de Investigaciones Sociológicas (CIS), Spain.
