# Supplementary material for: Positioning analysis of Spanish politicians through their Twitter posts versus Spanish public opinion
Source: Humanit Soc Sci Commun. 2023 Jun 8;10(1):307. doi: 10.1057/s41599-023-01805-9 (PMC10248327; doi:10.1057/s41599-023-01805-9)
Supplement: Supplementary file 1 — Supplementary material_Table 3 [file 41599_2023_1805_MOESM1_ESM.docx]

**Table 3.** Column and row coordinate elements

| **Column** | **AXIS 1** | | |  | **AXIS 2** | | |
| --- | --- | --- | --- | --- | --- | --- | --- |
| **screen_name** | **Coordinate** | **Correlation** | **% Inertia explained** |  | **Coordinate** | **Correlation** | **% Inertia explained** |
| andoniortuzar | 0.05 | 0.001 | 0.02% |  | 0.226 | 0.024 | 0.77% |
| ArnaldoOtegi | 0.052 | 0.006 | 0.13% |  | 0.174 | 0.066 | 2.39% |
| gabrielrufian | -0.002 | 0 | 0.00% |  | 0.216 | 0.202 | 3.74% |
| ierrejon | -0.058 | 0.021 | 0.57% |  | 0.261 | 0.419 | 19.51% |
| InesArrimadas | 0.234 | 0.321 | 13.20% |  | -0.134 | 0.106 | 7.43% |
| ionebelarra | -0.233 | 0.395 | 14.90% |  | 0.135 | 0.133 | 8.56% |
| junqueras | -0.161 | 0.021 | 0.82% |  | 0.559 | 0.257 | 16.88% |
| KRLS | 0.108 | 0.056 | 1.37% |  | 0.15 | 0.108 | 4.50% |
| pablocasado_ | 0.289 | 0.527 | 25.15% |  | -0.077 | 0.037 | 3.05% |
| sanchezcastejon | -0.324 | 0.602 | 34.88% |  | -0.242 | 0.335 | 33.17% |
| Santi_ABASCAL | 0.328 | 0.365 | 8.99% |  | -0.005 | 0 | 0.00% |
| **Row** | **AXIS 1** | | |  | **AXIS 2** | | |
| **text** | **Coordinate** | **Correlation** | **% Inertia explained** |  | **Coordinate** | **Correlation** | **% Inertia explained** |
| today | -0.304 | 0.424 | 2.41% |  | -0.226 | 0.235 | 2.28% |
| government | 0.477 | 0.572 | 4.96% |  | -0.296 | 0.22 | 3.26% |
| country | -0.685 | 0.869 | 8.42% |  | -0.115 | 0.025 | 0.41% |
| spain | -0.014 | 0.001 | 0.00% |  | -0.408 | 0.791 | 4.74% |
| people | -0.342 | 0.546 | 1.65% |  | 0.175 | 0.143 | 0.74% |
| law | 0.316 | 0.24 | 1.20% |  | 0.085 | 0.017 | 0.15% |
| year | 0.065 | 0.047 | 0.05% |  | 0.171 | 0.328 | 0.64% |
| day | -0.071 | 0.011 | 0.06% |  | 0.344 | 0.269 | 2.28% |
| good | -0.079 | 0.027 | 0.08% |  | 0.148 | 0.096 | 0.46% |
| right | -0.106 | 0.024 | 0.12% |  | 0.395 | 0.339 | 2.97% |
| family | 0.021 | 0.002 | 0.01% |  | -0.224 | 0.213 | 0.94% |
| great | 0.059 | 0.01 | 0.04% |  | -0.313 | 0.288 | 1.80% |
| spanish | 0.258 | 0.171 | 0.66% |  | -0.206 | 0.109 | 0.72% |
| work | -0.328 | 0.485 | 1.03% |  | 0.032 | 0.005 | 0.02% |
| time | -0.317 | 0.453 | 0.91% |  | 0.143 | 0.092 | 0.32% |
| thank | -0.345 | 0.432 | 1.03% |  | 0.096 | 0.034 | 0.14% |
| freedom | 0.385 | 0.337 | 1.19% |  | 0.267 | 0.162 | 0.98% |
| support | -0.027 | 0.004 | 0.01% |  | -0.108 | 0.06 | 0.17% |
| more | -0.198 | 0.087 | 0.29% |  | 0.333 | 0.245 | 1.38% |
| congratulation | -0.006 | 0 | 0.00% |  | -0.251 | 0.221 | 0.82% |
