# Supplementary material for: Positioning analysis of Spanish politicians through their Twitter posts versus Spanish public opinion
Source: Humanit Soc Sci Commun. 2023 Jun 8;10(1):307. doi: 10.1057/s41599-023-01805-9 (PMC10248327; doi:10.1057/s41599-023-01805-9)
Supplement: Supplementary file 2 — Questionnaire [file 41599_2023_1805_MOESM2_ESM.pdf]

PUBLIC OPINION AND FISCAL POLICY STUDY. JULY 2021. CIS3332

"Information subject to statistical secrecy (Law 12/89, of 9 May, on the Public Statistical Function) and to the General Data Protection Regulation and the Organic Law on the Protection of Personal Data and Guarantee of Digital Rights". National Statistical Plan 2021-2024. RD 1110/2020, of 15 December.

"Good morning/afternoon, I am (my name) and I am conducting a special telephone survey for the Centro de Investigaciones Sociológicas (CIS) on topics of general interest. For this reason we ask for your cooperation and thank you in advance. This telephone number has been obtained at random. This conversation will be recorded for quality control and then deleted after less than one month. We guarantee the absolute anonymity and secrecy of your answers in strict compliance with the laws on statistical confidentiality and personal data protection. After completing the survey, your telephone number will be dissociated from any answers you may give, which will in turn be anonymised so that under no circumstances can they be associated with you. If you would like to know your data protection rights and further information, you can consult the website www.cis.es. Have you understood the information you have read and would you be so kind as to answer a few questions? The survey takes about 10 minutes. You are not obliged to answer all the questions. Thank you very much.

PC1. Question contact 1. Can you tell me which province and municipality I am calling...?

[TEL_TYPE].
FIXED 1
MOBILE 2

[CAAC] [CAAC

[PROVINCE] [PROVINCE

[MUNICIPALITY]

[CAPITAL]

[TAMUNI]

[INTERVIEW].

INTERVIEWER: IF THE PERSON WHO ANSWERS IS DIFFERENT FROM THE PERSON WHO PICKED UP THE PHONE INTRODUCE HIM/HERSELF:

Good morning/afternoon, my name is... and I am calling from the Centro de Investigaciones Sociológicas because we are conducting an opinion poll on topics of general interest. It lasts about 10 minutes. Would you be so kind as to collaborate with us?

[SEX].
Man 1
Woman 2

[EDADEXACT].

[AGE].
from 18 to 24 1
from 25 to 34 2
from 35 to 44 3
from 45 to 54 4
from 55 to 64 5
65 and over 6

Q.0 First of all, I would like to ask you if you have .....
[P0]
Spanish nationality 1
Spanish and other nationality 2
Other nationality 3

Jumps:
If P0=3 go to end of questionnaire.

P.1 How satisfied are you with these aspects of your life? Use a scale from 0 to 10, where 0 means "completely dissatisfied" and 10 means "completely satisfied".
[P1]

	0 Complet. dissatisfied	1	2	3	4	5	6	7	8	9	10 Complet. satisfech o/a	N.S.	N.C.
Your family life	0	1	2	3	4	5	6	7	8	9	10	98	99
Your health	0	1	2	3	4	5	6	7	8	9	10	98	99
Your social life	0	1	2	3	4	5	6	7	8	9	10	98	99
Their standard of living	0	1	2	3	4	5	6	7	8	9	10	98	99

Study: PUBLIC OPINION SURVEY AND FISCAL PCA. JUL.2021
CIS3332

P.2 In general terms, how happy or unhappy do you consider yourself to be? Please use a scale from 0 to 10, where 0 means that you consider yourself "completely unhappy" and 10 means that you consider yourself "completely happy".

[ESCAFELI].

- *0. Completely unhappy* 0
- 1 1
- 2 2
- 3 3
- 4 4
- 5 5
- 6 6
- 7 7
- 8 8
- 9 9
- *10. Completely happy* 10
- *N.S.* 98
- *N.C.* 99

P.3 Would you say that you can generally trust most people, or that you are never cautious enough in your dealings with others? Please place yourself on a scale of 0 to 10, where 0 means "never careful enough" and 10 means "most people can be trusted".

[SCACONFIANZA].

- *0. You can never be prudent enough* 0
- 1 1
- 2 2
- 3 3
- 4 4
- 5 5
- 6 6
- 7 7
- 8 8
- 9 9
- *10. Most people can be trusted* 10
- *N.S.* 98
- *N.C.* 99

P.4 When considering someone as a good citizen, how important do you consider each of the following attitudes and behaviours? Use a scale from 0 to 10, where 0 means "not at all important" and 10 means "very important".

[P4]

	0. Nothing important before	*1*	*2*	*3*	*4*	*5*	*6*	*7*	*8*	*9*	*10. Very important before*	*N.S.*	*N.C.*
**Be in solidarity with people who is worse off than you.**	0	1	2	3	4	5	6	7	8	9	10	98	99
**Voting in elections**	0	1	2	3	4	5	6	7	8	9	10	98	99
**No tax evasion**	0	1	2	3	4	5	6	7	8	9	10	98	99
**Always comply with laws and regulations rules**	0	1	2	3	4	5	6	7	8	9	10	98	99
**Respect the opinions of others others even if they are different from one's own**	0	1	2	3	4	5	6	7	8	9	10	98	99
**Be a responsible person and honest**	0	1	2	3	4	5	6	7	8	9	10	98	99

Q.5 I would like you to tell me how often, a lot, quite a lot, a little or not at all, do you talk to someone close to you, a family member or friend, a work colleague, about the functioning of public services?

[P5]

- *A lot* 1
- *Quite* 2
- *Little* 3
- *None* 4
- *N.S.* 8
- *N.C.* 9

Q.6 To what extent would you say that each of the following public services perform very, fairly, poorly or not at all satisfactorily?

[P6]

	Very satisfying.	*Quite satisfactory.*	*(DO NOT READ) Regular*	*Little satisfaction.*	*Nothing satisfactory.*	*N.S.*	*N.C.*
**Teaching**	1	2	3	4	5	8	9
**Health care**	1	2	3	4	5	8	9
**Pension management**	1	2	3	4	5	8	9
**The Administration of Justice**	1	2	3	4	5	8	9
**Citizen security**	1	2	3	4	5	8	9
**Social services**	1	2	3	4	5	8	9
**Public transport**	1	2	3	4	5	8	9
**Public works (roads, sewage treatment plants, etc.)**	1	2	3	4	5	8	9
**Helping people dependents**	1	2	3	4	5	8	9

Q.7 I would like you to tell me which of the following statements best reflects your views on taxation.

[P7]

Taxes are a means to better redistribution wealth in society *1*
Taxes are something the state forces us to pay. pay without really knowing in return for what *2*
Taxes are necessary for the state to can provide public services *3*
N.S. *8*
N.C. *9*

P.8 As you know, the different public administrations use the money we pay in taxes in Spain to finance the public services and benefits we have been talking about. Please tell me if you think that too much or too little money is spent on each of the services I am going to mention.

[P8]

	Too many	*The necessary*	*Very few*	*N.S.*	*N.C.*
**Teaching**	1	2	3	8	9
**Public works**	1	2	3	8	9
**Unemployment protection**	1	2	3	8	9
**Defence**	1	2	3	8	9
**Citizen security**	1	2	3	8	9
**Health**	1	2	3	8	9
**Culture**	1	2	3	8	9
**Housing**	1	2	3	8	9
**Justice**	1	2	3	8	9
**Social Security/Pensions**	1	2	3	8	9
**Transport and communications**	1	2	3	8	9
**Environmental protection**	1	2	3	8	9
**Development cooperation**	1	2	3	8	9
**Science and technology research**	1	2	3	8	9
**Aid to dependent persons**	1	2	3	8	9

P.9 Some people think that public services and social benefits should be improved, even if they have to pay more taxes (on a scale of 0 to 10, these people would rank 0). Others think it is more important to pay less tax, even if this means reducing public services and social benefits (they would rank 10 on the scale). Still others are somewhere in between. Where would you place yourself?

[ESCAIMPUESTOS].

0. Improve public services even if it is necessary to pay more taxes *0*
1 .... *1*
2 .... *2*
3 .... *3*
4 .... *4*
5 .... *5*
6 .... *6*
7 .... *7*
8 .... *8*
9 .... *9*
10. Pay less tax even if taxes have to be reduced public services *10*
N.S. *98*
N.C. *99*

P.10 In general, taking into account existing public services and social benefits, would you say that, on the whole, society benefits a lot, quite a lot, a little or not at all from what we pay to public administrations in taxes and contributions?

[P10]

A lot *1*
Quite *2*
Little *3*
Nothing *4*
N.S. *8*
N.C. *9*

P.11 And more specifically, taking into account what you and your family receive from the different public administrations, would you say that the public administrations give you more than what you pay in taxes and contributions, more or less than what you pay, or less than what you pay in taxes and contributions?

[P11]

More than you pay *1*
About what you pay *2*
Less than you pay *3*
N.S. *8*
N.C. *9*

P.12 Would you say that what Spaniards pay in taxes is too much, too little or too much?

[P12]

A lot *1*
Regular *2*
Little *3*
N.S. *8*
N.C. *9*

Q.13 And in comparison with other more advanced European countries, do you think that Spain pays more, the same or less taxes?

[P13]

More taxes 1
Equal taxation 2
Less taxes 3
(DO NOT READ) Don't know, can't compare with others countries 8
N.C. 9

Q.14 In your opinion taxes should be collected:

[P14]

Especially with direct taxes, such as personal income tax (according to the income or wealth of individuals and companies) 1
Especially with indirect taxes, such as VAT (which affects everyone equally) (they are levied on consumer goods). consumption and services) 2
N.S. 8

Q.15 And do you think that, in general, taxes are levied fairly, that is, that those who have more pay more, or don't you think so?

[P15]

Yes, they are fairly charged 1
He does not think so 2
N.S. 8
N.C. 9

Q.16 Do you think that Spaniards, when it comes to paying taxes, are very conscious and responsible, quite conscious and responsible, not very conscious or not at all conscious and responsible?

[P16]

Highly aware and responsible 1
Quite aware and responsible 2
Lack of awareness and responsibility 3
Neither conscious nor responsible 4
N.S. 8
N.C. 9

Q.17 Do you personally consider yourself to be very conscious and responsible, quite conscious and responsible, not very conscious and responsible or not very conscious and responsible at all?

[P17]

Highly aware and responsible 1
Quite aware and responsible 2
Little awareness and responsibility 3
Nothing conscious or responsible 4
N.C. 9

Q.18 In your opinion, do you think that in Spain there is a lot of tax fraud, quite a lot, a little or very little tax fraud?

[P18]

There is a lot of tax fraud 1
There is a lot of tax fraud 2
There is little tax fraud 3
There is very little tax fraud 4
N.S. 8
N.C. 9

Q.19 Among the people you know, how many of them do you think actually declare all their income when filing their personal income tax returns: all or almost all, a lot, a little or none?

[P19]

All or almost all 1
Quite 2
Little 3
None 4
N.S. 8
N.C. 9

Q.20 And among the people you know who are obliged to declare VAT (professionals, self-employed, etc.), How much do you think you actually declare all your income when you do your VAT return: all or almost all, a lot, a little or none?

[P20]

All or almost all 1
Quite 2
Little 3
None 4
N.S. 8
N.C. 9

Q.21 Among the following, what do you think is the main reason why people hide part or all of their income from the tax authorities? (IWER: READ ONCE AND THEN REPEAT READING).
Reasons for concealing income from the tax authorities

[P21A]

Lack of control by the administration 1
Penalties that can be imposed are minor, 'it is cheap to defraud'. 2
The shortage of jobs forces people to accept any kind of work even if such income is not declared 3
Wages are too low and we have to look for other options for raising money 4
Taxes to be paid to the Treasury are excessive 5
Lack of honesty and civic awareness 6
Because those who have the most avoid (or minimise) the payment of taxes 7
Another answer, which one? 96
N.S. 98
N.C. 99

Filters:
If NO P21A=(96) go to next.

Q.22 Which of the following do you think is the main effect of tax fraud? (IWER: READ ONCE, THEN REPEAT READING).

[P22]

In general, it does not really have an effect important 1
Decreases resources to fund services public services and social benefits 2
Forces an increase in the tax burden on those who comply and pay their taxes correctly 3
It discourages those who pay their taxes correctly 4
It creates injustices, as some have to pay what they have to pay for. stop paying others 5
N.S. 8
N.C. 9

P.23 Now I would like to tell me whether you agree or disagree with each of the following sentences:
[P23]

	I rather agree	*Rather disagree*	*N.S.*	*N.C.*
**If people are no longer cheating the tax authorities, it is because they are afraid of a review**	1	2	8	9
**Almost everyone cheats a little when paying their taxes, and the Administration already has this in place**	1	2	8	9
**It is actually not wrong to hide part of the income, because it does not harm anyone.**	1	2	8	9
**Cheating the Treasury is cheating the rest of the citizens.**	1	2	8	9

P.24 Do you think that the government is currently making too much, too little, too much or too little effort to combat tax fraud?

Q24A And for explaining the destination of taxes?
[P24]
- *Many*........1
- *Quite a few*........2
- *Few*........3
- *Very few*........4
- *N.S.*........8
- *N.C.*........9

[P24A]
- *Many*........1
- *Quite a few*........2
- *Few*........3
- *Very few*........4
- *N.S.*........8
- *N.C.*........9

P.25 What would you say is the general VAT rate applied in Spain: 7%, 16%, 21% or 27%? (IWER: "General VAT rate" is the default rate applied to all products and services: household appliances, clothes, footwear, health products, petrol, etc., different from reduced VAT, which applies to food, transport, hotels, restaurants and shows, and from super-reduced VAT, which applies to basic necessities: bread, milk, books, etc.).
[VAT].
- *7% of the total*........1
- *16% of*........2
- *21% of the total*........3
- *27% of*........4
- *N.S.*........8
- *N.C.*........9

P.26 I am going to read you a series of sentences. Tell me to what extent you consider each of the following behaviours to be very tolerable, fairly tolerable, not very tolerable or not tolerable at all:
[P26]

	Very tolerable	*Fairly tolerable*	*(DO NOT READ) Depends*	*Poorly tolerable*	*Nothin g tolerable*	*N.S.*	*N.C.*
**Failure to declare all income for income tax purposes (IRPF)**	1	2	3	4	5	8	9
**Paying without invoice for a domestic repair to avoid paying VAT**	1	2	3	4	5	8	9
**Applying a deduction that you are not entitled to when making the payment for taxes (VAT or personal income tax return)**	1	2	3	4	5	8	9
**Receiving a social benefit to which you are not entitled (faking an illness to get a sick leave or to receive a social security benefit). unemployment benefit when paid work is carried out)**	1	2	3	4	5	8	9
**Being self-employed and not charging VAT**	1	2	3	4	5	8	9
**Be self-employed and deduct personal expenses (clothes, purchase of the supermarket, etc.) as business expenses that do not apply to you.**	1	2	3	4	5	8	9
**For a large company to avoid or avoid paying corporation tax**	1	2	3	4	5	8	9
**For a small business to avoid or evade paying the tax on the companies**	1	2	3	4	5	8	9
**Setting up an Internet-only business to pay less taxes**	1	2	3	4	5	8	9

P.27 And do you think that, in general, these types of behaviours described above could be tolerated to some extent, or could they not be tolerated by...?
[P27]

	They could be tolerated	*They could not be tolerated*	*(DO NOT READ) Depends on what type, person, magnitude, etc.*	*N.S.*	*N.C.*
**Your neighbours**	1	2	3	8	9
**Your friends**	1	2	3	8	9
**Your family environment**	1	2	3	8	9

P.28 Have you filed a tax return for the current year (for 2020 income)?

[RENT].

- *Yes* ... 1
- *No* ... 2
- *No obligation to do so* ... 3
- *N.C.* ... 9

Q.28A Would you mind telling me whether your tax return was positive (payable) or negative (refundable)?

[RESULT] [RESULT

- *Positive (payable)* ... 1
- *Negative (to be returned)* ... 2
- *Neither payable nor returnable* ... 3
- *N.C.* ... 9

Q.28B Could you please tell me if you have ticked the box that allocates a percentage of your taxes: to the Catholic Church, to social purposes, to both, or not at all?

[BOX].

- *To the Catholic Church* ... 1
- *For social purposes* ... 2
- *To both (Catholic Church and social purposes)* ... 3
- *No allocation/No box ticked* ... 4
- *N.S./N.R.* ... 8
- *N.C.* ... 9

Q.28C Do you consider it very likely, fairly likely, not very likely or not at all likely that your tax return will be revised?

[REVIEW].

- *Very likely* ... 1
- *Quite likely* ... 2
- *Unlikely* ... 3
- *Not likely* ... 4
- *N.S.* ... 8
- *N.C.* ... 9

P.29 In your opinion, and at the present time, you would say that Spain is a country...

[INEQUALITY]

- *Where there are few social inequalities* ... 1
- *Where there are some significant inequalities in some respects, but not in others* ... 2
- *Where there are large inequalities in general* ... 3
- *N.S.* ... 8
- *N.C.* ... 9

P.30 Could you tell me which of the following options you agree with the most? (INTERVIEWER: READ ONCE AND THEN REPEAT READING).

[INTERVENED].

- *The state should not intervene in economic life, should be left to private initiative* ... 1
- *The state should not intervene in economic life, except to correct possible misalignments* ... 2
- *The state must intervene in economic life, but respect private initiative.* ... 3
- *The state must intervene in all economic life.* ... 4
- *N.S.* ... 8
- *N.C.* ... 9

P.31 Some people think that people's economic position depends almost exclusively on their effort, education and professional worth (on a scale of 0 to 10 they would be placed at 0). Others think that what really matters is family background, contacts or simply luck (these would be placed at point 10). What do you think most influences the economic position people achieve in Spain?

[ESCAPE].

- *0. Effort, education and professional worth* ... 0
- *1* ... 1
- *2* ... 2
- *3* ... 3
- *4* ... 4
- *5* ... 5
- *6* ... 6
- *7* ... 7
- *8* ... 8
- *9* ... 9
- *10. Family background, contacts or luck* ... 10
- *N.S.* ... 98
- *N.C.* ... 99

P.32 Assuming that general elections are held again tomorrow, i.e. to the Spanish Parliament, which party would you vote for (SPONTANEOUS ANSWER, DO NOT READ ANSWER CHOICES!!!).

[INTENTIONG]

- *PSOE (Spanish Socialist Workers' Party)* ... 2
- *PP (People's Party)* ... 1
- *VOX* ... 18
- *We can* ... 3
- *IU (United Left)* ... 5
- *United We Can* ... 21
- *En Comú Podem* ... 6
- *En Común - United We Can* ... 67
- *Citizens* ... 4
- *More Country* ... 50
- *ERC (Esquerra Republicana de Catalunya)* ... 8
- *JxCat (Junts per Catalunya)* ... 9
- *CUP* ... 19
- *EAJ-PNV (Basque Nationalist Party)* ... 11
- *EH Bildu (Euskal Herria Bildu)* ... 12
- *CC-PNC (Canary Islands Coalition - Nationalist Party) Canary Islands)* ... 13
- *New Canary Islands* ... 16
- *UPN (Unión del Pueblo Navarro)* ... 14
- *Compromís* ... 7
- *BNG (Galician Nationalist Bloc)* ... 24
- *PRC (Regionalist Party of Cantabria)* ... 43
- *Teruel Exists* ... 68
- *PACMA (Animal Party)* ... 17
- *FAC (Asturias Forum)* ... 15
- *Another match, which one?* ... 95
- *Null vote* ... 77
- *Blank* ... 96
- *I would not vote* ... 97
- *Don't know yet* ... 98
- *N.C.* ... 99

Filters:
If NO INTENTIONG=(95) go to next.

Q.32a If for any reason you did not vote for the party you told me about, which other party would you vote for? (SPONTANEOUS ANSWER, DO NOT READ THE ANSWER OPTIONS!!!).

[INTENCIONGALTER]

PSOE (Spanish Socialist Workers' Party)	*2*
PP (People's Party)	*1*
VOX	*18*
We can	*3*
IU (United Left)	*5*
United We Can	*21*
En Comú Podem	*6*
En Común - United We Can	*67*
Citizens	*4*
More Country	*50*
ERC (Esquerra Republicana de Catalunya)	*8*
JxCat (Junts per Catalunya)	*9*
CUP	*19*
EAJ-PNV (Basque Nationalist Party)	*11*
EH Bildu (Euskal Herria Bildu)	*12*
CC-PNC (Coalición Canaria - Partido Nacionalista Canario)	*13*
New Canary Islands	*16*
UPN (Unión del Pueblo Navarro)	*14*
Compromís	*7*
BNG (Nationalist Galician Bloc)	*24*
PRC (Regionalist Party of Cantabria)	*43*
Teruel Exists	*68*
PACMA (Animal Party)	*17*
FAC (Asturias Forum)	*15*
(DO NOT READ) I would not vote for any other party (cite the same party)	*93*
Another match, which one?	*95*
Null	*77*
Blank	*96*
I would not vote	*97*
N.S.	*98*
N.C.	*99*

Filters:
If NO INTENTIONGALTER=(95) go to next.

P.33 Without any commitment on your part, could you tell me which party you feel most sympathy for (SPONTANEOUS ANSWER, DO NOT READ ANSWER OPTIONS!!!).

[SIMPATHY].

PSOE (Spanish Socialist Workers' Party)	*2*
PP (People's Party)	*1*
VOX	*18*
We can	*3*
IU (United Left)	*5*
United We Can	*21*
En Comú Podem	*6*
En Común - United We Can	*67*
Citizens	*4*
More Country	*50*
ERC (Esquerra Republicana de Catalunya)	*8*
JxCat (Junts per Catalunya)	*9*
CUP	*19*
EAJ-PNV (Basque Nationalist Party)	*11*
EH Bildu (Euskal Herria Bildu)	*12*
CC-PNC (Canary Islands Coalition - Nationalist Party) Canary Islands)	*13*
New Canary Islands	*16*
UPN (Unión del Pueblo Navarro)	*14*
Compromís	*7*
BNG (Galician Nationalist Bloc)	*24*
PRC (Regionalist Party of Cantabria)	*43*
Teruel Exists	*68*
PACMA (Animal Party)	*17*
FAC (Asturias Forum)	*15*
Another match, which one?	*95*
None	*97*
N.S.	*98*
N.C.	*99*

Filters:
If NO SIMPATIA=(95) go to the next one.

P.34 When talking about politics, the expressions left and right are often used. On a scale of 1 to 10, where 1 means "left" and 10 means "right", in which box would you place yourself?

[ESCIDEOL]

1 Left	*1*
2	*2*
3	*3*
4	*4*
5	*5*
6	*6*
7	*7*
8	*8*
9	*9*
10 Right.	*10*
(DO NOT READ) None	*97*
N.S.	*98*
N.C.	*99*

P.35 Can you tell me if in the general election on 10 November 2019 you will be able to vote in the general election on 10 November 2019? (READ).

[PARTICIPATIONG]
[PARTICIPATIONG

Went to vote and voted	*1*
Postal vote	*7*
Not old enough to vote	*2*
Went to vote but was unable to do so	*3*
Didn't go to vote because he couldn't	*4*
He preferred not to vote	*5*
Did not have the right to vote	*6*
No recollection	*8*
N.C.	*9*

Q.35A And could you tell me which party or coalition you voted for (SPONTANEOUS ANSWER).

Filters:
If NO PARTICIPATIONG=(1;7) go to the next one.

[RECUVOTOG]

PSOE......2
PP......1
VOX......18
United We Can......21
En Comú Podem......6
En Común - United We Can......67
Citizens......4
More Country......50
ERC......8
JxCat......9
CUP......19
EAJ-PNV......11
EH Bildu......12
CCa-PNC-NC......13
Navarra Suma (UPN)......14
Més Compromís......7
BNG (Galician Nationalist Bloc)......24
PRC (Regionalist Party of Cantabria)......43
Teruel Exists......68
PACMA (Animal Party)......17
Other matches......95
Blank......96
Null vote......77
No recollection......98
N.C.......99

P.36 What is your marital status?

ECIVIL] [ECIVIL

Married......1
Single......2
Widowed......3
Separated......4
Divorced......5
N.C.......9

P.37 What is your current living situation, i.e. are you living...?

[SITCONVIVEN] [SITCONVIVEN

Alone......1
Alone with your child/children (with or without other relatives)......2
With your husband or wife or partner with children (with or without other relatives or family members)......3
With husband/wife or childless partner (with or without other relatives or family members)......4
With their father and/or mother with or without siblings (with or without siblings) without other relatives or family members)......5
Another situation......6
N.C.......9

P.38 Have you ever been to school or attended any kind of education (IWER: if no, ask if you can read and write).

[SCHOOL].

No, he/she is illiterate......1
No, but can read and write......2
Yes, he went to school......3
N.C.......9

Q.38A What is the highest level of education that you have completed, i.e. you have the corresponding official qualification? Please specify as much as possible, telling me the course and the name of the course when you completed it: (e.g. 3 years of primary school, primary school, 5th baccalaureate, industrial master, pre-university, EGB, bachelor, doctorate, FP1, etc.) (IWER: if you are still studying, please write down the last course you have completed and the correct cycle in the answer choices. If you have not completed primary school, write down the number of years you attended school).

[COURSEENTREV].

COURSE _________ *N.S. - N.R. = 98* *N.C. = 99*

[NAMEISENTREV].

NAME OF FIRM _________ *N.S. - N.R. = 98* *N.C. = 99*

[LEVELSTENTREV]

01. Less than 5 years of schooling......1
02. Primary education (LOGSE Primary Education, 5th grade of EGB, Old Primary Education)......2
03. Initial Vocational Qualification (IVVET). PCPI (Initial Vocational Qualification Programmes, which do not require an academic qualification from the first stage of secondary education. Social guarantee programmes......3
04. Secondary education (ESO, EGB. School Graduate. School Certificate, Baccalaureate Elementary)......4
05. Intermediate vocational training (VET cycle/module (intermediate level), Plastic Arts and Design, Music and Dance, Sports Education, VET I, Vocational Baccalaureate) elementary. Industrial Officer; Commercial Baccalaureate......5
06. Baccalaureate (Bachillerato LOGSE, BUP, Higher Baccalaureate (6th year), University Baccalaureate (7th), Including COU and PREU)......6
07. VET of higher grade (VET cycle/training module (higher grade) of Plastic Arts, Design, Music and Dance, Sport, VET II, Bach. Industrial Master's Degree, Industrial Master's Degree, Mercantile Expert; 2nd grade secretarial training; Intermediate Conservatory......7
08. Architecture-technical engineering (Architecture/engineering, Quantity Surveyor; Surveyors)......8
09. Diploma (ATTENTION: only official diplomas, do not code here the first three years of the first degree) a bachelor's or master's degree of a longer duration).......9
10. Degree (Bachelor's degree, equivalent Artistic Education (since 2006)......10
11. Degree (Qualifications with official equivalence: 2nd cycle INEF; Dance and Drama (since 1992); Higher grade of music)......11
12. Architecture/engineering......12
13. Official university master's degree (Specialities medical or equivalent)......13
14. PhD......14
15. Own postgraduate degrees (non-official master's degrees, etc.)......15
16. Other studies......16
N.S.......98
N.R.......99

P.39 How do you define yourself in religious matters: practising Catholic, non-practising Catholic, believer in another religion, agnostic, indifferent or non-believer, or atheist?

[RELIGION].

- *Practising Catholic* 1
- *Non-practising Catholic* 2
- *Believer of another religion* 3
- *Agnostic (does not deny the existence of God but They don't rule it out either)* 4
- *Indifferent, non-believer* 5
- *Atheist (denies the existence of God)* 6
- *N.C.* 9

P. 9A How often do you attend mass or other religious services, excluding occasions related to social ceremonies, e.g. weddings, communions or funerals?

Filters:
If NO RELIGION=(1;2;3) go to the next one.

- *Never* 1
- *Almost never* 2
- *Several times a year* 3
- *Two or three times a month* 4
- *Every Sunday and public holidays* 5
- *Several times a week* 6
- *N.C.* 9

P.40 How would you rate your personal financial situation at present: very good, good, fair, poor, bad or very poor?

[SITECONOM]

- *Very good* 1
- *Good* 2
- *Regular* 3
- *Mala* 4
- *Very bad* 5
- *N.S.* 8
- *N.C.* 9

P.41 Which of the following situations are you currently in?

SITLAB] [SITLAB

- *Works* 1
- *Retired or pensioner (previously worked)* 2
- *Pensioner (not previously employed)* 3
- *Unemployed and has worked before* 4
- *Unemployed and looking for his first job* 5
- *Student* 6
- *Unpaid domestic work (previously unpaid) has worked)* 7
- *Unpaid domestic work (previously unpaid) has worked)* 8
- *Other situation, which one?* 9
- *N.C.* 99

Filters:
If NO SITLAB=(9) go to the next one.

P.42 Can you tell me what your current occupation is (READ).

Filters:
If NO SITLAB=1 go to the next one.

[CNO11]

- *Directors and managers* 1
- *Professionals and scientists and intellectuals* 2
- *Technicians and mid-level professionals* 3
- *Administrative support staff* 4
- *Service workers and shop and market sellers* 5
- *Farmers and skilled agricultural, forestry and fishery workers* 6
- *Craft and skilled trades workers, craftsmen and craftswomen mechanical and other trades* 7
- *Plant and machine operators and assemblers* 8
- *Elementary occupations* 9
- *Military occupations and police forces* 10
- *Other* 11
- *N.C.* 99

P.43 Do you work as... (READ).

Filters:
If NO SITLAB=1 go to the next one.

RELALAB] [RELALAB

- *Permanent employee (salaried, commission, daily wage, etc.), on a fixed basis)* 1
- *Temporary or interim employee (on salary, commission, daily wage, etc., on a temporary or interim basis)* 2
- *Employer or professional with employees* 3
- *Professional or self-employed (without employees)* 4
- *Family allowance (without regulated remuneration in a family member's company or business)* 5
- *Member of a cooperative* 6
- *Other situation, which one?* 7
- *N.C.* 9

Filters:
If NO RELALAB=(7) go to the next one.

P.44 What social class would you say you belong to? (SPOT ANSWER, DO NOT READ ANSWER CHOICES).

[CLASESOCIAL CLASS].

- *High class* 1
- *Upper-middle class* 2
- *Middle-middle class* 3
- *Lower-middle class* 4
- *Working/working class* 5
- *Low class* 12
- *Poor class* 6
- *Infraclass* 7
- *Proletariat* 8
- *To those at the bottom* 9
- *Excluded* 10
- *To ordinary people* 11
- *Other (specify)* 96
- *Does not believe in classes* 97
- *Don't know, doubt* 98
- *N.C.* 99

Filters:
If NO SOCIAL CLASS=(96) go to the next one.

WE HAVE FINISHED. THANK YOU VERY MUCH FOR YOUR KINDNESS AND FOR THE TIME YOU HAVE SPENT WITH US.
DEDICATED
